# Baroreflex Sensitivity Measured by Pulse Photoplethysmography

**DOI:** 10.3389/fnins.2019.00339

**Published:** 2019-04-18

**Authors:** Jesús Lázaro, Eduardo Gil, Michele Orini, Pablo Laguna, Raquel Bailón

**Affiliations:** ^1^Department of Biomedical Engineering, University of Connecticut, Storrs, CT, United States; ^2^Biomedical Signal Interpretation and Computational Simulation (BSICoS) Group, Aragón Institute of Engineering Research (I3A), IIS Aragón, University of Zaragoza, Zaragoza, Spain; ^3^Centro de Investigación Biomédica en Red en Bioingeniería, Biomateriales y Nanomedicina (CIBER-BBN), Madrid, Spain; ^4^Department of Mechanical Engineering, University College London, London, United Kingdom

**Keywords:** baroreflex, photoplethysmography, alpha index, autonomic nervous system, blood pressure, cardiovascular assessment

## Abstract

Novel methods for assessing baroreflex sensitivity (BRS) using only pulse photoplethysmography (PPG) signals are presented. Proposed methods were evaluated with a data set containing electrocardiogram (ECG), blood pressure (BP), and PPG signals from 17 healthy subjects during a tilt table test. The methods are based on a surrogate of α index, which is defined as the power ratio of RR interval variability (RRV) and that of systolic arterial pressure series variability (SAPV). The proposed α index surrogates use pulse-to-pulse interval series variability (PPV) as a surrogate of RRV, and different morphological features of the PPG pulse which have been hypothesized to be related to BP, as series surrogates of SAPV. A time-frequency technique was used to assess BRS, taking into account the non-stationarity of the protocol. This technique identifies two time-varying frequency bands where RRV and SAPV (or their surrogates) are expected to be coupled: the low frequency (LF, inside 0.04–0.15 Hz range), and the high frequency (HF, inside 0.15–0.4 Hz range) bands. Furthermore, time-frequency coherence is used to identify the time intervals when the RRV and SAPV (or their surrogates) are coupled. Conventional α index based on RRV and SAPV was used as Gold Standard. Spearman correlation coefficients between conventional α index and its PPG-based surrogates were computed and the paired Wilcoxon statistical test was applied in order to assess whether the indices can find significant differences (*p* < 0.05) between different stages of the protocol. The highest correlations with the conventional α index were obtained by the α-index-surrogate based on PPV and pulse up-slope (PUS), with 0.74 for LF band, and 0.81 for HF band. Furthermore, this index found significant differences between rest stages and tilt stage in both LF and HF bands according to the paired Wilcoxon test, as the conventional α index also did. These results suggest that BRS changes induced by the tilt test can be assessed with high correlation by only a PPG signal using PPV as RRV surrogate, and PPG morphological features as SAPV surrogates, being PUS the most convenient SAPV surrogate among the studied ones.

## 1. Introduction

The baroreflex system plays an important role in regulating short-term fluctuations of arterial blood pressure (BP) (La Rovere et al., 2008; Robertson et al., 2012). Arterial baroreceptors (placed in the wall of the carotid sinuses and aortic arch) sense changes in BP and modulate efferent autonomic neural activity to the central nervous system accordingly. A rise in sensed BP leads to an increase of vagal neurons discharge and a decrease in the discharge of sympathetic neurons, resulting in decreased heart rate (HR), cardiac contractility and peripheral vascular resistance. On the contrary, decreased BP enhances sympathetic and inhibits vagal activity, leading to increased HR, cardiac contractility and peripheral vascular resistance.

Cardiovascular diseases are frequently associated to an impairment of baroreflex mechanisms, resulting in chronic adrenergic activation. Reduced baroreflex control of HR has been reported in coronary artery disease, heart failure, hypertension and myocardial infarction (La Rovere et al., 2008; Pinna et al., 2017). Assessment of baroreflex in humans is usually approached measuring the changes in HR in response to changes in BP, the so-called baroreflex sensitivity (BRS). Alternatively, spontaneous beat-to-beat fluctuations of systolic arterial pressure and RR interval can be analyzed, allowing BRS assessment during daily-life. A wide spectrum of techniques has been used for spontaneous beat-to-beat BRS assessment. Traditional approaches, such as the sequence technique and those based on the spectral analysis of systolic arterial pressure and RR interval series (α index), were reviewed in La Rovere et al. (2008).

In order to deal with the nonstationary nature of cardiovascular variability, methods based on wavelet transform (Nowak et al., 2008; Keissar et al., 2010) and quadratic time-frequency representations (Orini et al., 2011) have been proposed. In Orini et al. (2012) a framework for nonstationary BRS assessment, based on a time-frequency distribution, was presented, taking into account the strength and prevalent direction of local coupling between RR variability (RRV) and systolic arterial pressure variability (SAPV) series. Alternatively, in Chen et al. (2011) dynamic assessment of BRS is accomplished based on a closed loop model within a point process framework. A critical review of clinical studies using spontaneous BRS was reported in Pinna et al. (2017). Despite some limitations, such as the lack of standards and the poor measurability in some patient populations, published studies support spontaneous BRS as a powerful tool for prognostic prediction in diseases such as hypertension, myocardial infarction, chronic heart failure and diabetes (La Rovere et al., 2008; Di Rienzo et al., 2009; de Moura-Tonello et al., 2016).

Spontaneous BRS assessment and monitoring during daily life is limited by the requirement of continuous BP recording, which is usually accomplished by the volume-clamp method or tonometry method, neither of them being suitable for ubiquitous monitoring (Mukkamala et al., 2015). This limitation may be overcome by using a surrogate of systolic arterial pressure which does not require the BP recording. Many works have attempted BP estimation based on pulse transit time (PTT), which is the time delay for the pressure wave to travel between two arterial sites. Most of these approaches, reviewed in Mukkamala et al. (2015), are based on models of arterial wall mechanics and wave propagation in the artery. Due to ease of measurement, pulse arrival time (PAT), which is the time delay between the electrocardiogram (ECG) waveform and a distal arterial waveform, has been widely used instead of PTT for BP estimation. PAT is the sum of PTT and the pre-ejection period (PEP), which varies beat-to-beat depending on ventricular and arterial pressures, short-term physiologic control and medication. Although the effect of PEP modulation makes PAT more inconvenient than PTT for BP estimation, half of the studies reviewed in Mukkamala et al. (2015) used PAT as a surrogate of PTT. Some of these methods have been used for BRS assessment. For instance, in Abe et al. (2015) it was proposed to evaluate baroreflex function using the maximum normalized cross-correlation between the LF components of HRV and PAT, derived from ECG and pulse photoplethysmographic (PPG) signals.

In Liu et al. (2011) it was suggested that PAT can track BP variations in HF range, but was inadequate to follow the LF variations. To overcome this limitation (Ding et al., 2016) proposed to estimate BP combining PAT with a new index, the photoplethysmogram intensity ratio (PIR), which can reflect changes in arterial diameter due to arterial vasomotion. In order to avoid PEP influence in BP estimation, PTT has been derived from impedance plethysmography recorded at the wrist and PPG at the finger (Huynh et al., 2018), or from a ballistocardiogram and PPG at the foot (Martin et al., 2016). Alternatively, PTT was estimated from two PPG signals recorded at ear and toe in Chen et al. (2009) and at forearm and wrist in Wang et al. (2018). Some works have investigated the correlation between PAT and PTT estimated from PPG signals at finger and forehead at rest (Liu et al., 2015) and during a tilt-test (Lázaro et al., 2016). In Li et al. (2014) different PPG indices were investigated for BP estimation. The time ratio of systole to diastole, time span of PPG cycle, diastolic time duration and area ratio of systole to diastole are at least as good as PTT for BP estimation, and can be derived from just one PPG signal.

The PPG signal can be acquired with a sensor placed in many places of the body. Furthermore, its recording is very simple, economical, and comfortable for the subject. Thus, PPG signal is a very interesting signal for ambulatory scenarios and wearable devices, and assessing BRS from PPG signal may have significant impact in such applications. Moreover, several studies have compared pulse rate variability (PRV), derived from the PPG to HRV derived from the ECG, reporting good agreement even in non-stationary situations and during abrupt autonomic nervous system changes (Gil et al., 2010; Wong et al., 2012; Posada-Quintero et al., 2013; Schfer and Vagedes, 2013). In this work we investigate the feasibility of assessing BRS solely from one PPG signal. The proposed approach is based on using PPG-based surrogates of RRV and SAPV series. On one hand, pulse-to-pulse variability (PPV) series was used as surrogate of RRV series. On the other hand, different PPG morphological features which are hypothesized to be related to the BP were used for generating series that were used as surrogates of SAPV. The ability of the proposed methods to capture changes in autonomic nervous system control was evaluated in a tilt-test database.

## 2. Materials and Methods

### 2.1. Data and Preprocessing

A data set containing ECG, BP, and PPG recordings from 17 healthy subjects (11 men), aged 28.5 ± 2.5 years, during a tilt table test was used for method evaluation. The protocol started with 4 min in supine position (Rest1), followed with 5 min in 70°-tilt-up position (Tilt), and ended with 4 min back to supine position (Rest2). The table took 18 s for automatic transitions between stages.

ECG lead V4 was recorded by Biopac ECG100C with a sampling rate of 1,000 Hz, BP signal (*x*_BP_(*n*)) was recorded by Finometer system with a sampling rate of 250 Hz, and PPG signal was recorded from the index finger by BIOPAC OXY100C with a sampling rate of *F*_*s*_ = 250 Hz. A low-pass filter with a cut-off frequency of 35 Hz was applied to the PPG in order to attenuate noise. This preprocessed PPG signal is denoted *x*_PPG_(*n*) in this paper. Several points were measured over the PPG pulses. Some of them were measured directly over the pulse as those described in section 2.1.1, and others over the waves extracted from the pulse by the pulse decomposition analysis (PDA) technique described in section 2.1.2.

#### 2.1.1. Pulse Delineation

**Figure 1:**
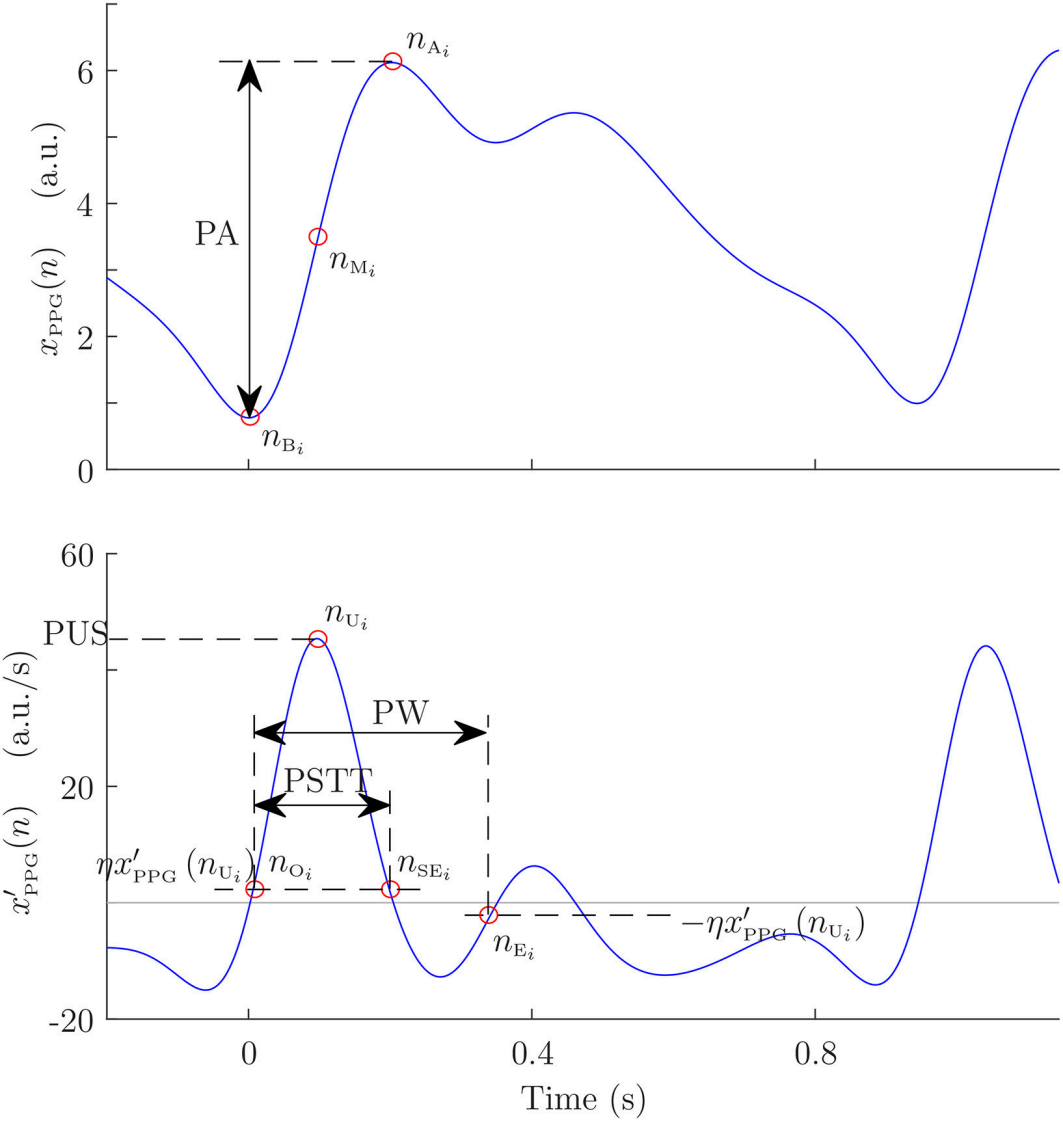
Example of PPG pulse with its automatically detected points, and morphological measures taken from them: pulse amplitude (PA), pulse width (PW), and pulse slope transit time (PSTT).

Several points of the *i*th PPG pulse were detected in order to take different morphological measurements. All these points are illustrated in Figure 1. First, PPG pulses were detected by an algorithm based on a low-pass derivative and a time-varying threshold (Lázaro et al., 2014). This algorithm detects the maximum up-slope point (*n*_U_*i*__), and later it is used for detecting the pulse apex point (*n*_A_*i*__) and the pulse basal point (*n*_B_*i*__) as:

(1) $$\begin{array}{lll} n_{{\text{A}}_{i}} & = & \underset{n}{\text{arg max}}\left\{x_{\text{PPG}}\left(n\right)\right\},\;n\in \left[n_{{\text{U}}_{i}},n_{{\text{U}}_{i}}+0.3F_{s}\right] \end{array}$$

(2) $$\begin{array}{lll} n_{{\text{B}}_{i}} & = & \underset{n}{\text{arg min}}\left\{x_{\text{PPG}}\left(n\right)\right\},\;n\in \left[n_{{\text{U}}_{i}}-0.3F_{s},n_{{\text{U}}_{i}}\right]. \end{array}$$

Subsequently, *n*_A_*i*__ and *n*_B_*i*__ are used to compute the medium-amplitude point (*n*_M_*i*__). This point is considered as a robust measure of PPG pulse location because it is located during the interval of the steepest slope of the PPG pulse, and it is set as:

(3) $$\begin{array}{l} n_{{\text{M}}_{i}}=\underset{n}{\text{arg min}}\left\{|x_{\text{PPG}}\left(n\right)-\frac{x_{\text{PPG}}\left(n_{{\text{A}}_{i}}\right)+x_{\text{PPG}}\left(n_{{\text{B}}_{i}}\right)}{2}|\right\},\\ \;n\in \left[n_{{\text{B}}_{i}},n_{{\text{A}}_{i}}\right]. \end{array}$$

Pulse onset *n*_O_*i*__ and end *n*_E_*i*__ points were detected based on the first derivative (Lázaro et al., 2013). In addition, pulse up-slope end *n*_SE_*i*__ was detected in a similar way. Let $x_{\text{PPG}}^{\prime }\left(n\right)$ be the first derivative of *x*_PPG_(*n*) computed by successive differences, after a 5-Hz-low-pass filter. Then, *n*_SE_*i*__ is set as:

(4) $$\begin{array}{l} n_{{\text{SE}}_{i}}=\underset{n}{\text{arg min}}\left\{|x_{\text{PPG}}^{\prime }\left(n\right)-\eta x_{\text{PPG}}^{\prime }\left(n_{{\text{U}}_{i}}\right)|\right\},\;n\in \left[n_{{\text{U}}_{i}},n_{{\text{A}}_{i}}\right], \end{array}$$

where η was set to 0.05 similarly to the case of *n*_O_*i*__ and *n*_E_*i*__ (Lázaro et al., 2013).

#### 2.1.2. Pulse Decomposition Analysis

PDA is a field in PPG signal proccessing that consits of modeling the PPG pulse as a main wave superposed with several reflected waves, increasing the robustness of some morphological measurements and even allowing others that would not be possible directly over the pulse. Several models can be found in the literature, based on different shapes including Gaussians (Baruch et al., 2011), LogNormal (Huotari et al., 2011), and Rayleight (Goswami et al., 2010). In this work, a modification to the PDA technique presented in Lázaro et al. (2018) is proposed. The main difference of this technique with respect to other PDA techniques in the literature is that the waves are extracted one-by-one, instead of fitting a several-waves-model at once. The modification proposed in this paper consists of not assuming a specific shape for the superposed waves, although it is assumed that they are symmetrical.

First, the baseline of the PPG signal was estimated by cubic-spline-interpolation of *x*_PPG_(*n*_B_*i*__), and subsequently subtracted from *x*_PPG_(*n*). This baseline-removed version of PPG signal is denoted $x_{\text{PPG}}^{\text{b}}\left(n\right)$ in this manuscript. Then, the beginning and the end of the *i*th PPG pulse were considered to be *n*_B_*i*__ and *n*_B_*i*+1__, respectively. Note that this criterion ensures that each PPG pulse begins and ends with zero amplitude, as subtracted baseline was estimated at those *n*_B_*i*__. Later, the algorithm extracts recursively the *j*th inner wave of the pulse by the following steps:

1. Set the beginning of the up-slope of the *j*th wave ($n_{{\text{SO}}_{j,i}}^{\text{b}}$) as the previous to the first non-zero-amplitude sample. Note that in case of *j* = 1 (the main wave), this corresponds to *n*_B_*i*__.
2. Set the end of the up-slope of the *j*th wave ($n_{{\text{SE}}_{j,i}}^{\text{b}}$) as the first relative maximum.
3. Estimate the *j*th wave $y_{j,i}^{\text{b}}\left(n\right)$ by concatenating the up-slope with itself horizontally flipped, assuming that it is symmetric:
  (5) $$\begin{array}{lll} x_{j,i}^{\text{b}}\left(n\right) & = & \{\begin{array}{cc} x_{\text{PPG}}^{\text{b}}\left(n\right), & n\in \left[n_{{\text{SO}}_{j,i}}^{\text{b}},n_{{\text{SE}}_{j,i}}^{\text{b}}\right]\\ 0, & \text{otherwise} \end{array} \end{array}$$
  (6) $$\begin{array}{lll} y_{j,i}^{\text{b}}\left(n\right) & = & x_{j,i}^{\text{b}}\left(n\right)+x_{j,i}^{\text{b}}\left(-n+2n_{{\text{SE}}_{j,i}}^{\text{b}}+1\right), \end{array}$$
4. Substract $y_{j,i}^{\text{b}}\left(n\right)$ to $x_{\text{PPG}}^{\text{b}}\left(n\right)$ and go back to step 1 to continue extracting the (*j* + 1)th wave.

**Figure 2:**
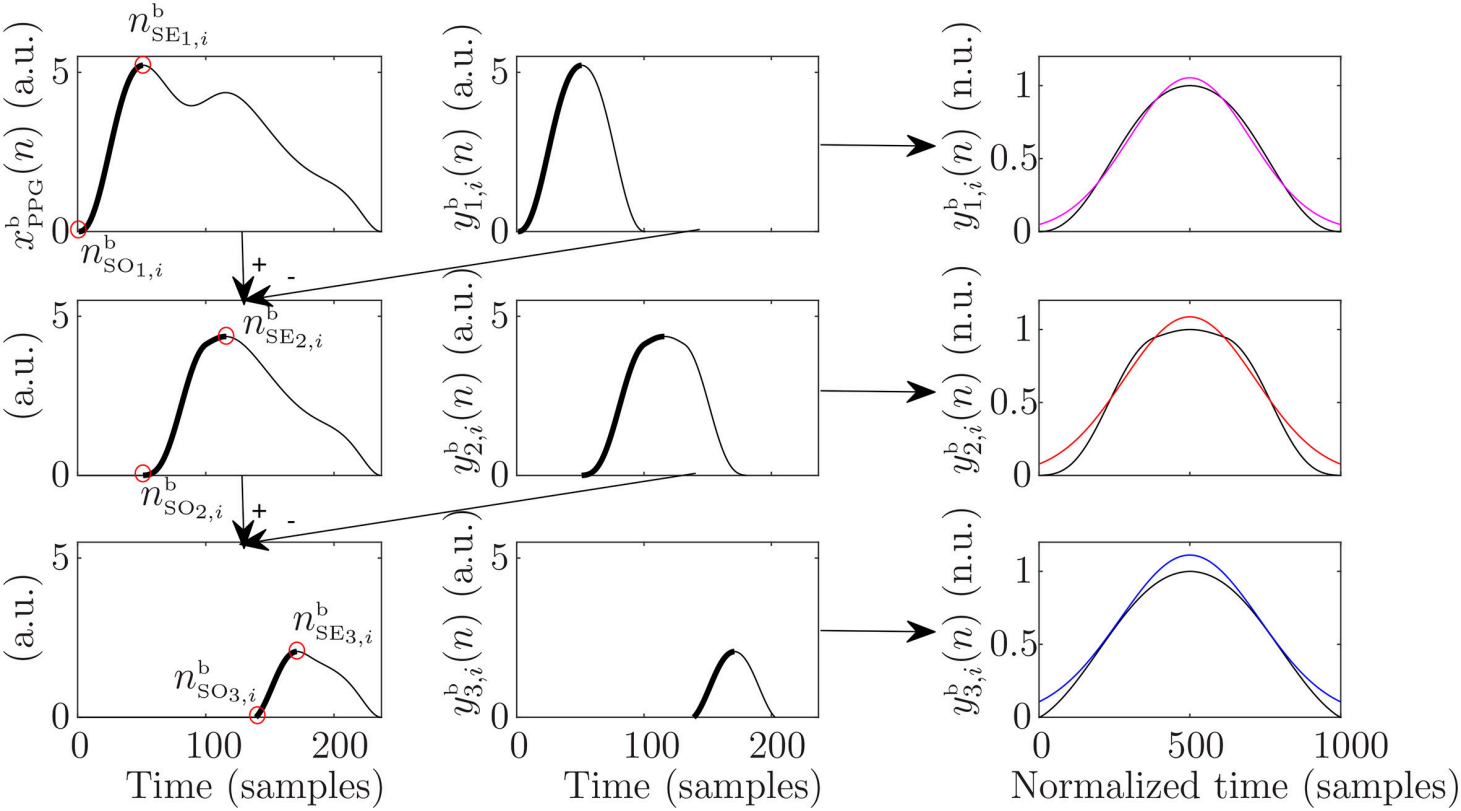
Example of the steps for the pulse decompostion analysis that lead to extraction of main wave (first row), first reflected wave (second row), and second reflected wave (third row). In addition, the subsequent modeling of the extracted waves can be observed in the third column in magenta, red, and blue for the main, first reflected, and second reflected waves, respectively.

Once the desired number of waves have been extracted, they can be modeled in order to measure morphological features. In this work, three waves were extracted per PPG pulse. Subsequently, these $y_{j,i}^{\text{b}}\left(n\right)$ were normalized to the unit in amplitude and to 1,000 samples by spline interpolation, and then they were modeled as Gaussian waves, each one defined by an amplitude, a mean, and a standard deviation (SD). Once these values are estimated, they were re-converted to the original scales of amplitude and time. An illustration of the steps of this algorithm can be observed in Figure 2.

### 2.2. PPG-Based Surrogates of Systolic Arterial Pressure Variability for BRS Estimation

#### 2.2.1. Systolic Arterial Pressure Variability Surrogates Based on Pulse Signal

Four pulse morphological features that have been related to the BP and/or to the arterial stiffness in the literature were measured from each PPG pulse: amplitude (PA), width (PW), up-slope (PUS), and slope transit time (PSTT). Pulse amplitude and width were measured as in Lázaro et al. (2013). The pulse amplitude corresponds to that amplitude reached by *n*_A_*i*__ with respect to *n*_B_*i*__, and the pulse width was measured as the time interval between *n*_O_*i*__ and *n*_E_*i*__. Pulse up-slope was measured as the first derivative value at *n*_U_*i*__, and PSTT was measured as the time interval between *n*_O_*i*__ and *n*_SE_*i*__. Later, PA, PW, PUS, and PSTT series were computed as:

(7) $$\begin{array}{lll} d_{\text{PA}}^{u}\left(n\right) & = & \underset{i}{\sum }\left[x_{\text{PPG}}\left(n_{{\text{A}}_{i}}\right)-x_{\text{PPG}}\left(n_{{\text{B}}_{i}}\right)\right]\delta \left(n-n_{{\text{M}}_{i}}\right) \end{array}$$

(8) $$\begin{array}{lll} d_{\text{PW}}^{u}\left(n\right) & = & \underset{i}{\sum }\left[n_{{\text{E}}_{i}}-n_{{\text{O}}_{i}}\right]\delta \left(n-n_{{\text{M}}_{i}}\right) \end{array}$$

(9) $$\begin{array}{lll} d_{\text{PUS}}^{u}\left(n\right) & = & \underset{i}{\sum }\left[x_{\text{PPG}}^{\prime }\left(n_{{\text{U}}_{i}}\right)\right]\delta \left(n-n_{{\text{M}}_{i}}\right) \end{array}$$

(10) $$\begin{array}{lll} d_{\text{PSTT}}^{u}\left(n\right) & = & \underset{i}{\sum }\left[n_{{\text{SE}}_{i}}-n_{{\text{O}}_{i}}\right]\delta \left(n-n_{{\text{M}}_{i}}\right), \end{array}$$

where δ(·) denotes the Kronecker delta function, and the superscript “*u*” denotes that the signals are unevenly sampled, as the PPG pulses occur unevenly in time. A median-absolute-deviation outlier-rejection (Bailón et al., 2006) rule was applied to each one of these series, rejecting those points of the series that are outside the boundaries defined as the median ± 5 times the SD of the previous 50 points. Subsequently, a 4-Hz-evenly sampled version of each one of them was obtained by linear interpolation. The resulting signals are denoted using the same nomenclature, this time without the superscript “*u*” [e.g., *d*_PA_(*n*)].

#### 2.2.2. Systolic Arterial Pressure Variability Surrogates Based on Pulse Decomposition Analysis

Seven morphological features were extracted from each PDA-based modeled PPG pulse. Specifically, the amplitude, mean, and twice the SD of the Gaussian-function fitted to the main wave were studied (*m*_A1_, *m*_B1_, and *m*_C1_, respectively). Moreover, the feature related to twice the SD of the first reflected wave were also studied (*m*_C2_), as well as the time delay between the main wave occurrence *m*_B1_ and those of reflected ones *m*_B2_ and *m*_B3_ (*m*_T12_ and *m*_T13_, respectively). Furthermore, the percentage of amplitude that it is lost in the first reflection was also estimated as:

(11) $$\begin{array}{l} m_{\text{A12}}=\frac{m_{\text{A1}}-m_{\text{A2}}}{m_{\text{A1}}}. \end{array}$$

**Figure 3:**
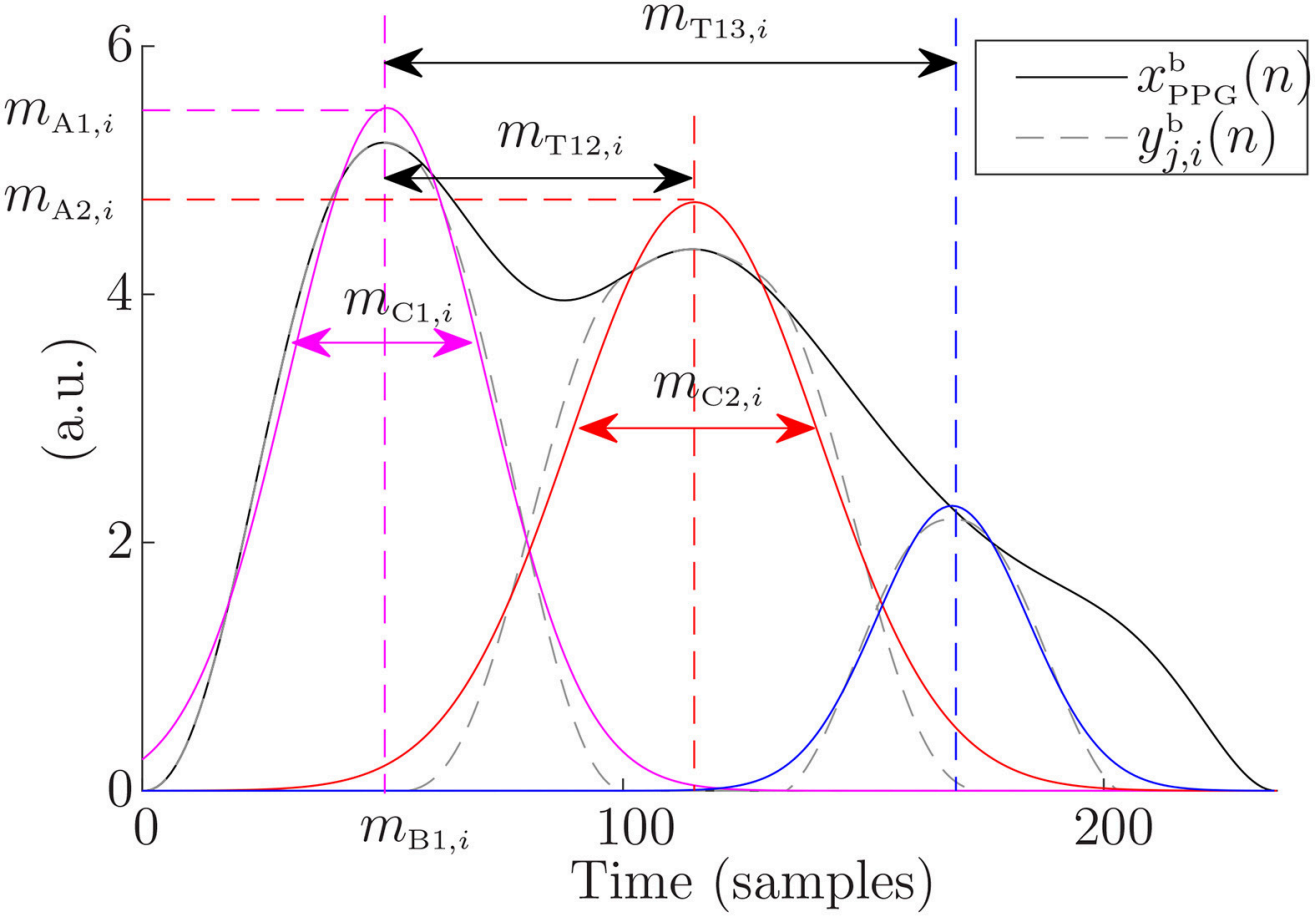
Measures over the extracted waves of an example PPG pulse. Gaussians fitted to these waves are shown in color.

Figure 3 illustrate these measures. These features extracted from the PDA are also hypothesized to be related to the BP and/or to the arterial stiffness since they are related to amplitude, relative position between the waves, and waves dispersion by SD. Their associated series were computed as:

(12) $$\begin{array}{l} d_{\left\{\text{A1, B1, C1, C2, T12, T13, A12}\right\}}^{u}\left(n\right)=\underset{i}{\sum }m_{\left\{\text{A1, B1, C1, C2, T12, T13, A12}\right\}}\delta \left(n-n_{{\text{B}}_{i}}\right). \end{array}$$

The outliers of these series were rejected by the same median-absolute-deviation-based rule applied in the case of the features which were measured over the pulse (see section 2.2.1), and similarly, they were linearly interpolated obtaining a 4 Hz evenly sampled version of each one of them denoted without the superscript “*u*”.

### 2.3. Baroreflex Sensitivity Indices

The BRS indices were computed based on the α index, which is computed from a spectral analysis of RRV and SAPV. Several α-index surrogates based on PPG signal were computed, using PPV as RRV surrogate, and the SAPV surrogates described above.

The PPV was estimated using *n*_M_*i*__ as fiducial point:

(13) $$\begin{array}{l} d_{\text{PPV}}^{u}\left(n\right)=\underset{i}{\sum }\frac{1}{F_{s}}\left[n_{{\text{M}}_{i}}-n_{{\text{M}}_{i-1}}\right]\delta \left(n-n_{{\text{M}}_{i}}\right). \end{array}$$

These series were also outlier-rejected and linearly interpolated to an even sampling rate of 4 Hz. Then, a power spectrum was computed from *d*_PPV_(*n*), obtaining ${\bar{S}}_{\text{PPV}}\left(f\right)$, and from the *k*th SAPV surrogates, obtaining *S*_*k*_(*f*), for each one of the stages of the protocol, where *k* can be PA, PW, PUS, PSTT, A1, B1, C1, C2, T12, T13, and A12. These power spectra were obtained by the Welch periodogram, using a 2 min Hamming window and 50% of overlap. Then, the PPG-based surrogates of the α index were extracted from these spectra, within LF ([0.04, 0.15] Hz) and HF ([0.15, 0.4] Hz) bands:

(14) $$\begin{array}{l} \alpha_{k}^{\left\{\text{LF},\text{HF}\right\}}=\sqrt{\int_{\Omega_{\left\{\text{LF},\text{HF}\right\}}}S_{\text{PPV}}\left(f\right)df/\int_{\Omega_{\left\{\text{LF},\text{HF}\right\}}}S_{k}\left(f\right)df}, \end{array}$$

where Ω_LF_ and Ω_HF_ denote the LF and HF bands, respectively.

In addition, in order to take into account the non-stationarity of the protocol, the BRS indices were computed using a time-frequency technique for instantaneous measurement of α index, described in Orini et al. (2012). A time-frequency distribution was applied to *d*_PPV_(*n*) obtaining *S*_PPV_(*n, f*), and to each one of the PPG-morphology series used as SAPV surrogates obtaining *S*_*k*_(*n, f*). In addition, a cross time-frequency spectrum *S*_PPV,*k*_(*n, f*) was also computed as in Orini et al. (2012). The instantaneous frequencies of the main components of *S*_PPV,*k*_(*n, f*) within [ 0.04, 0.15] Hz [for LF band, *f*_LF_(*n*)] and [0.15, 0.4] Hz [for HF band *f*_HF_(*n*)] were computed as the frequencies where *S*_PPV,*k*_(*n, f*) is maximum within those bands. Then, Ω_LF_(*n*) and Ω_HF_(*n*) were defined as the frequency bands centered at *f*_LF_(*n*) and *f*_HF_(*n*), respectively, with a bandwidth equal to the frequency resolution of the used time-frequency distribution. Then, the PPG-based surrogate of α index was computed for each *S*_*k*_(*n, f*) as the square root of the ratio between the powers of *d*_PPV_(*n*) (as a surrogate of RRV) and *d*_*k*_(*n*), for each one of the defined bands:

(15) $$\begin{array}{l} \alpha_{k}^{\left\{\text{LF},\text{HF}\right\}}\left(n\right)=\sqrt{\int_{\Omega_{\left\{\text{LF},\text{HF}\right\}}}S_{\text{PPV}}\left(n,f\right)df/\int_{\Omega_{\left\{\text{LF},\text{HF}\right\}}}S_{k}\left(n,f\right)df}. \end{array}$$

**Figure 4:**
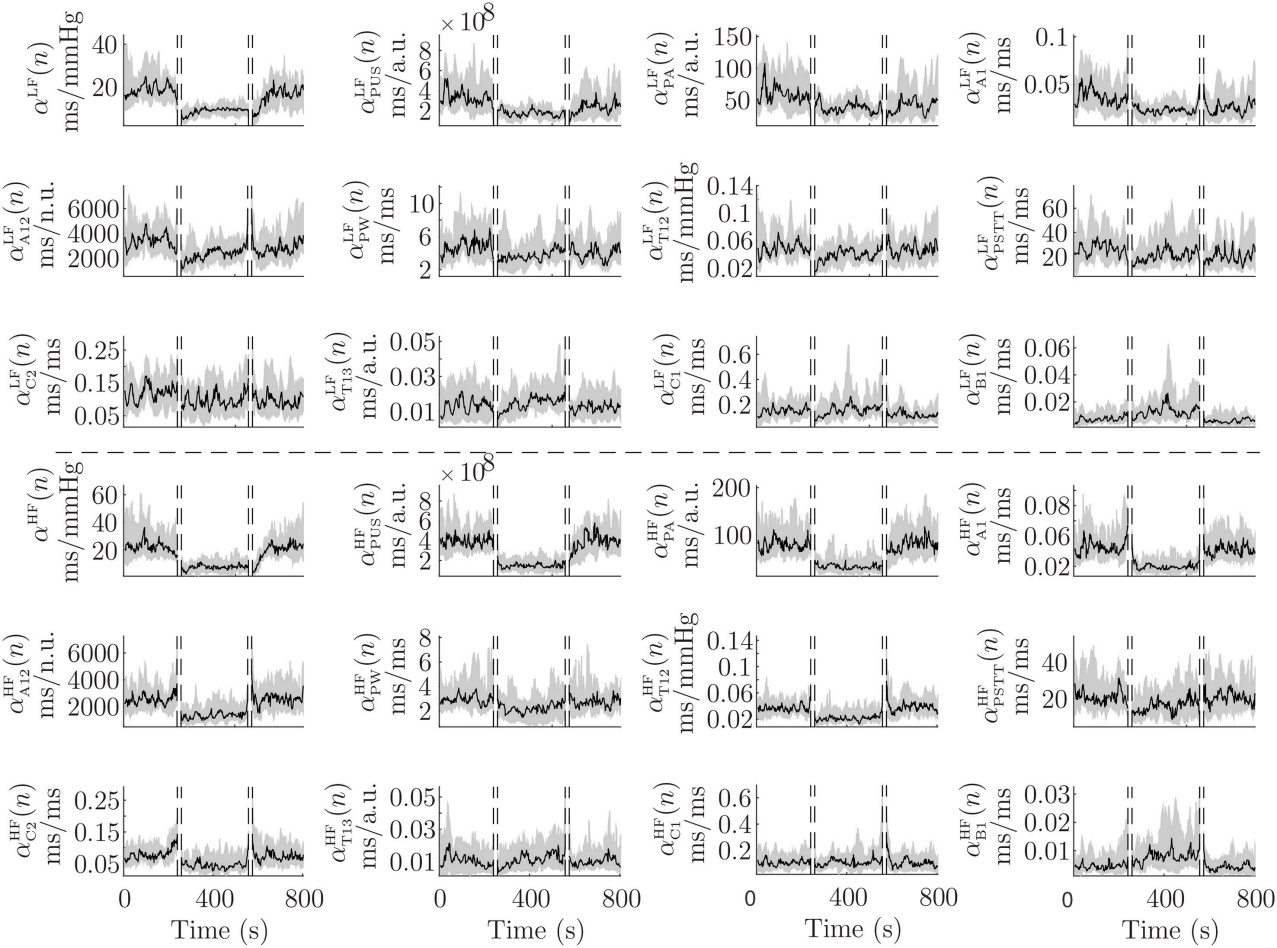
Median (black) and IQR (shaded area) of α^LF^(*n*), α^HF^(*n*), and their PPG-based surrogates, during the whole protocol. First 3 rows are related to the LF band and last 3 rows are related to the HF band.

Figure 4 shows inter-subject median and interquartile range (IQR) of $\alpha_{k}^{\left\{\text{LF},\text{HF}\right\}}\left(n\right)$ during the protocol. For BRS assessment, it is convenient to measure these indices only when PPV and *k* series are coupled. In order to detect these time courses, a time-frequency coherence (γ_PPV,*k*_(*n, f*)) was computed, and PPV and *k* series were considered to be coupled in those areas where γ_PPV,*k*_(*n, f*) is over a significance level. The indices $\alpha_{k}^{\text{LF}}\left(n\right)$ and $\alpha_{k}^{\text{HF}}\left(n\right)$ measured only when γ_PPV,*k*_(*n, f*) is significant within Ω_LF_ and Ω_HF_, respectively, are denoted $\alpha_{k}^{{\text{LF}}_{\gamma }}\left(n\right)$ and $\alpha_{k}^{{\text{HF}}_{\gamma }}\left(n\right)$, respectively, in this paper. Further details are given in Orini et al. (2012).

For validation purposes, the conventional α index was also computed from the RRV and the SAPV, denoted with no subindex [$\alpha^{\left\{LF,HF,LF_{\gamma },HF_{\gamma }\right\}}\left(n\right)$], and taken as reference. The RRV was computed by the interval function using the R points (*n*_R_*i*__) determined from the ECG by Martínez et al. (2004):

(16) $$\begin{array}{l} d_{\text{RRV}}^{u}\left(n\right)=\underset{i}{\sum }\frac{1}{F_{s}}\left[n_{{\text{R}}_{i}}-n_{{\text{R}}_{i-1}}\right]\delta \left(n-n_{{\text{R}}_{i}}\right). \end{array}$$

The SAPV was computed from the maximum of BP pulses (${\overset{⌣}{n}}_{{\text{A}}_{i}}$), which were detected similarly to the case of the PPG pulses (see section 2.1.1):

(17) $$\begin{array}{l} d_{\text{SAPV}}^{u}\left(n\right)=\underset{i}{\sum }x_{\text{BP}}\left({\overset{⌣}{n}}_{{\text{A}}_{i}}\right)\delta \left(n-{\overset{⌣}{n}}_{{\text{A}}_{i}}\right). \end{array}$$

### 2.4. Performance Metrics

A unique value per subject and stage of the protocol (Rest1, Tilt, and Rest2) was obtained for each one of the three studied α-index estimation methods:

1. Welch-periodogram approach (α^{LF, HF}^): As it is based on a non-time-frequency technique, a unique value per subject and stage is available.
2. Time-frequency approach (${\bar{\alpha }}^{\left\{\text{LF},\text{HF}\right\}}$): The median of α^{LF, HF}^(*n*) within each stage and each subject was taken as the unique value per subject and stage.
3. Time-frequency-coherence approach (${\bar{\alpha }}^{\left\{LF_{\gamma },HF_{\gamma }\right\}}$): The median of $\alpha^{\left\{LF_{\gamma },HF_{\gamma }\right\}}\left(n\right)$ within each stage and each subject was taken as the unique value per subject and stage.

Then, correlation between the indices (α and α_*k*_) obtained from the 17 subjects and the 3 stages of the protocol (Rest1, Tilt, and Rest2) were computed. The distributions of these indices were found to be not normal by the Kolmogorov-Smirnov test. Thus, the Spearman's correlation coefficient was used. Furthermore, the Wilcoxon signed-rank test was applied to see if the indices can find significant (*p* < 0.05) differences between the different stages of the protocol.

As the SAPV surrogates, the α-index surrogates have different units and magnitude than classical α index. Thus, these surrogates cannot be directly compared to the classical α index, but their evolution can be compared. In order to do this, the relative variation of the α-index between consecutive stages was computed for each subject:

(18) $$\begin{array}{l} \Delta \alpha =\frac{\alpha_{\text{S2}}-\alpha_{\text{S1}}}{\alpha_{\text{S1}}}, \end{array}$$

where α_S1_ and α_S2_ represent the studied index within stages S1 and S2, respectively.

A linear regression of the α-index surrogates which obtained best results in terms of correlation (those based on PUS, as it can be observed in section 3) was performed, obtaining similar units than those of the conventional α index (ms/mmHg). This linear regression was performed in order to compare those indices in a Bland-Altman plot. In addition, a multiple linear regression was performed using all the studied α-index surrogates in order to study whether their information is complementary or redundant. The combined α-index surrogates are denoted ${\hat{\alpha }}^{\left\{\text{LF},\text{HF}\right\}}$ (Welch-periodogram approach), ${\hat{\bar{\alpha }}}^{\left\{\text{LF},\text{HF}\right\}}$ (time-frequency approach), and ${\hat{\bar{\alpha }}}^{\left\{{\text{LF}}_{\gamma },{\text{HF}}_{\gamma }\right\}}$ (time-frequency-coherence approach).

## 3. Results

**Figure 5:**
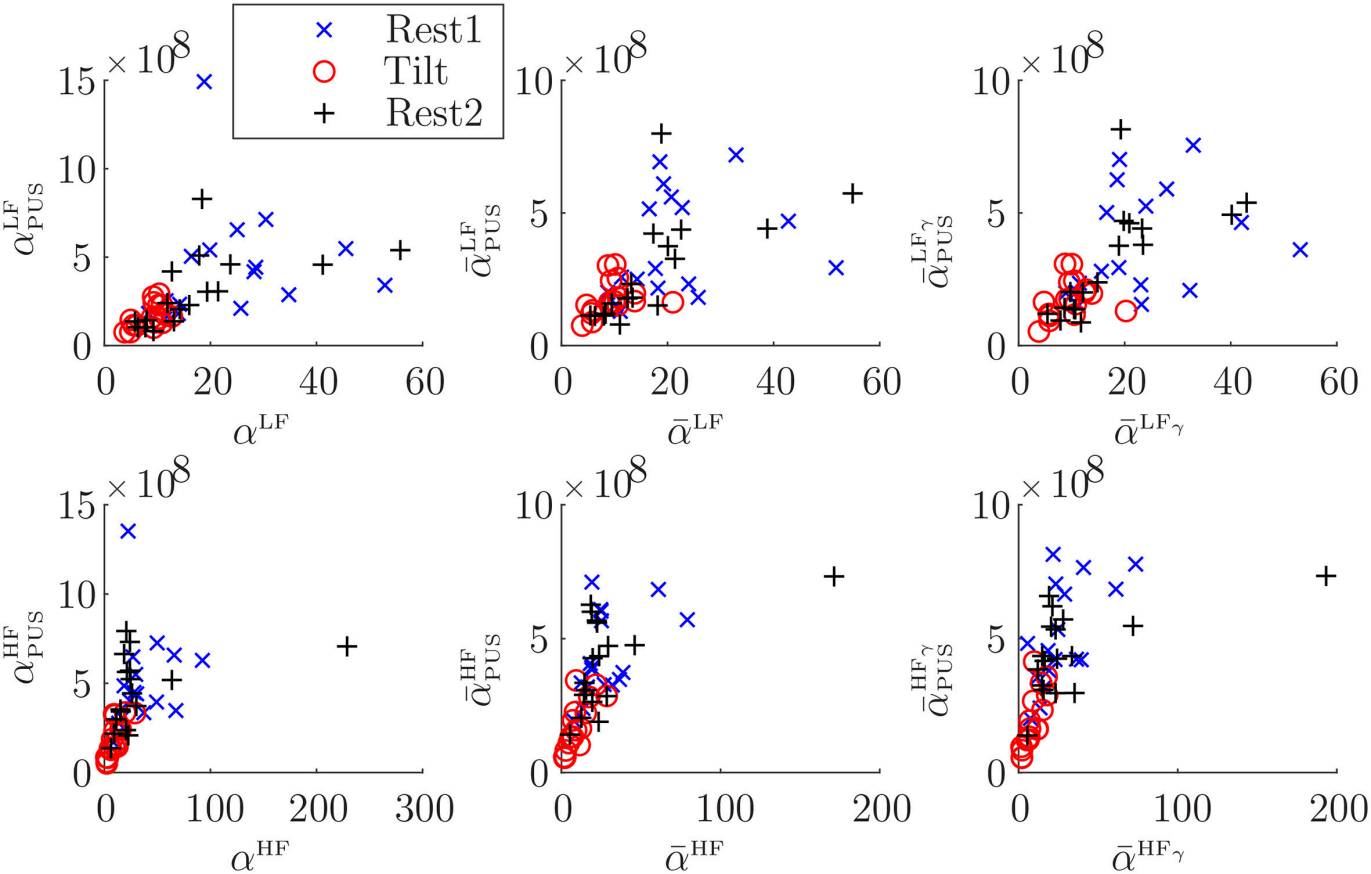
Scatterplots of α vs. α_PUS_ indices (first column), of $\bar{\alpha }$ vs. ${\bar{\alpha }}_{\text{PUS}}$ indices (second column), and of ${\bar{\alpha }}^{\gamma }$ vs. ${\bar{\alpha }}_{\text{PUS}}^{\gamma }$ indices (third column).

**Figure 6:**
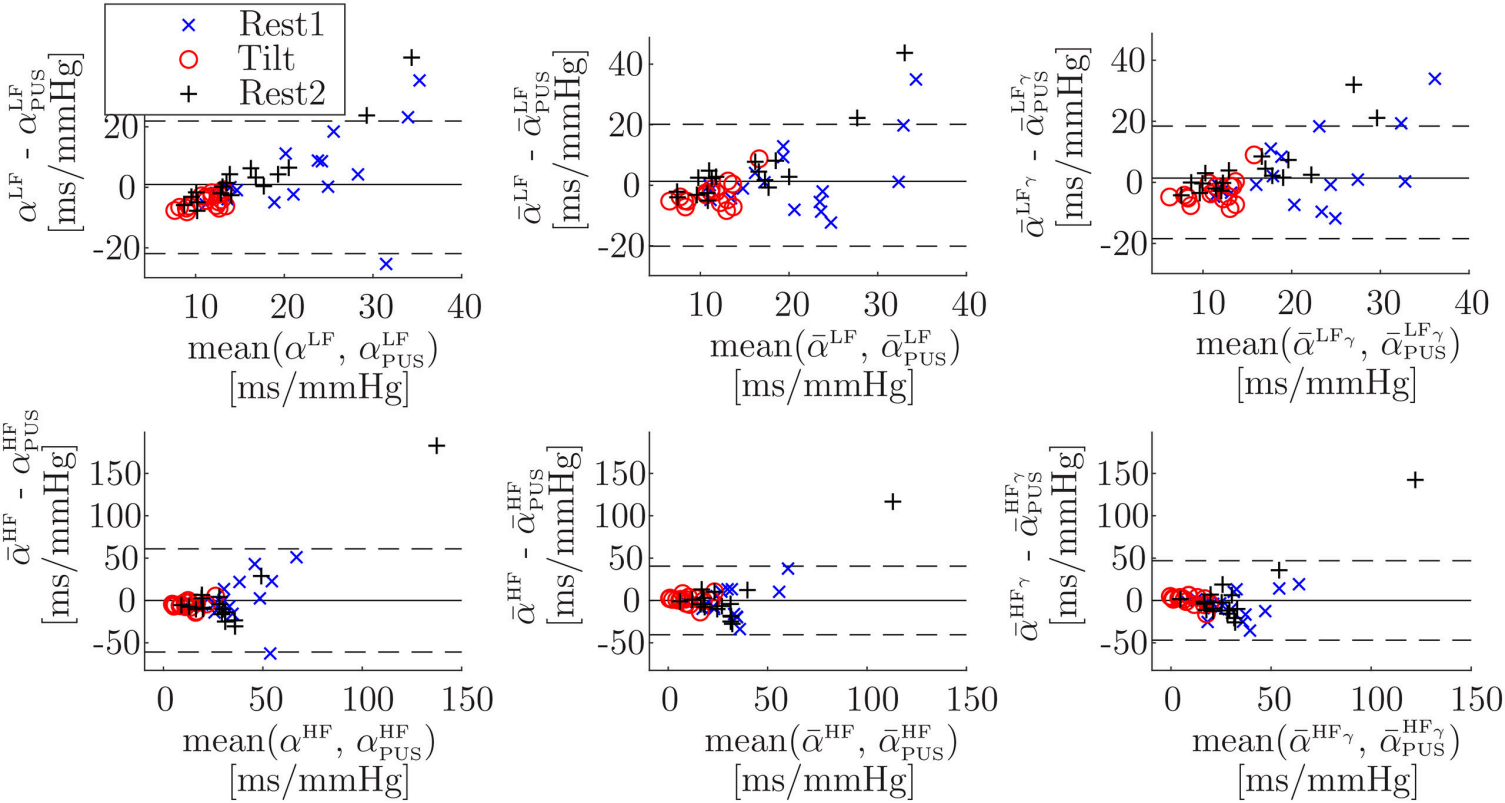
Bland-Altman plots of α vs. α_PUS_ indices (Welch-periodogram approach, first column), of $\bar{\alpha }$ vs. ${\bar{\alpha }}_{\text{PUS}}$ indices (time-frequency approach, second column), and of ${\bar{\alpha }}^{\gamma }$ vs. ${\bar{\alpha }}_{\text{PUS}}^{\gamma }$ indices (time-frequency coherence approach, third column), after a linear regression to convert all units to ms/mmHg. Note that scales are not the same for LF band (first row) than for HF band (second row).

Table 1 shows inter-subject Spearman's correlation coefficients between α^{LF, HF}^ and $\alpha_{k}^{\left\{\text{LF},\text{HF}\right\}}$, between ${\bar{\alpha }}^{\left\{\text{LF},\text{HF}\right\}}$ and ${\bar{\alpha }}_{k}^{\left\{\text{LF},\text{HF}\right\}}$, and between ${\bar{\alpha }}^{\left\{{\text{LF}}_{\gamma },{\text{HF}}_{\gamma }\right\}}$ and ${\bar{\alpha }}_{k}^{\left\{{\text{LF}}_{\gamma },{\text{HF}}_{\gamma }\right\}}$. The highest correlations were obtained for the α-index surrogates based on PUS. A scatterplot of these indices is shown in Figure 5. In addition, a Bland-Altmant plot of these indices and their associated conventional α indices is shown in Figure 6, after a linear regression in order to obtain similar units and magnitudes. The obtained limits of agreement were 0.94 ± 21.90 ms/mmHg (mean of the two values ± 1.96 × SD), –8.99E–15 ± 60.90, 1.29 ± 20.13, 4.56E–15 ± 40.49, 1.40 ± 18.43, 5.92E–15 ± 46.89 ms/mmHg, for $\alpha_{\text{PUS}}^{\text{LF}}$, $\alpha_{\text{PUS}}^{\text{HF}}$, ${\bar{\alpha }}_{\text{PUS}}^{\text{LF}}$, ${\bar{\alpha }}_{\text{PUS}}^{\text{HF}}$, ${\bar{\alpha }}_{\text{PUS}}^{{\text{LF}}_{\gamma }}$, and ${\bar{\alpha }}_{\text{PUS}}^{{\text{HF}}_{\gamma }}$, respectively.

**Table 1:** Inter-subject Spearman correlations of α^{LF, HF}^ and $\alpha_{k}^{\left\{\text{LF},\text{HF}\right\}}$ obtained in the different stages of the protocol.

	**α** **and** **α**_***k***_		$\bar{\alpha }$ **and** ${\bar{\alpha }}_{k}$		${\bar{\alpha }}^{\gamma }$ **and** ${\bar{\alpha }}_{k}^{\gamma }$	
**k**	**LF**	**HF**	**LF**	**HF**	**LF**	**HF**
PUS	0.81	0.80	0.74	0.76	0.74	0.81
A1	0.80	0.87	0.69	0.80	0.69	0.76
PA	0.77	0.79	0.67	0.79	0.67	–
A12	0.61	0.68	0.50	0.62	0.50	0.61
T12	0.48	0.53	0.48	0.61	0.48	0.56
PW	0.32	0.48	0.14	0.49	0.14	0.36
PSTT	0.48	0.36	0.39	0.27	0.39	0.29
C2	0.39	0.50	0.17	0.30	0.17	–
T13	0.20	0.18	0.14	0.10	0.14	–
C1	0.31	0.24	0.36	0.35	0.36	–
B1	0.03	0.02	0.10	0.05	0.10	–0.06

*In addition, inter-subject Spearman correlations of medians of ${\bar{\alpha }}^{\left\{\text{LF},\text{HF}\right\}}$ and ${\bar{\alpha }}_{k}^{\left\{\text{LF},\text{HF}\right\}}$ obtained in the different stages of the protocol are also shown, as well as the inter-subject Spearman correlations of medians of ${\bar{\alpha }}^{\left\{{\text{LF}}_{\gamma },{\text{HF}}_{\gamma }\right\}}$ and ${\bar{\alpha }}_{k}^{\left\{{\text{LF}}_{\gamma },{\text{HF}}_{\gamma }\right\}}$*.

**Figure 7:**
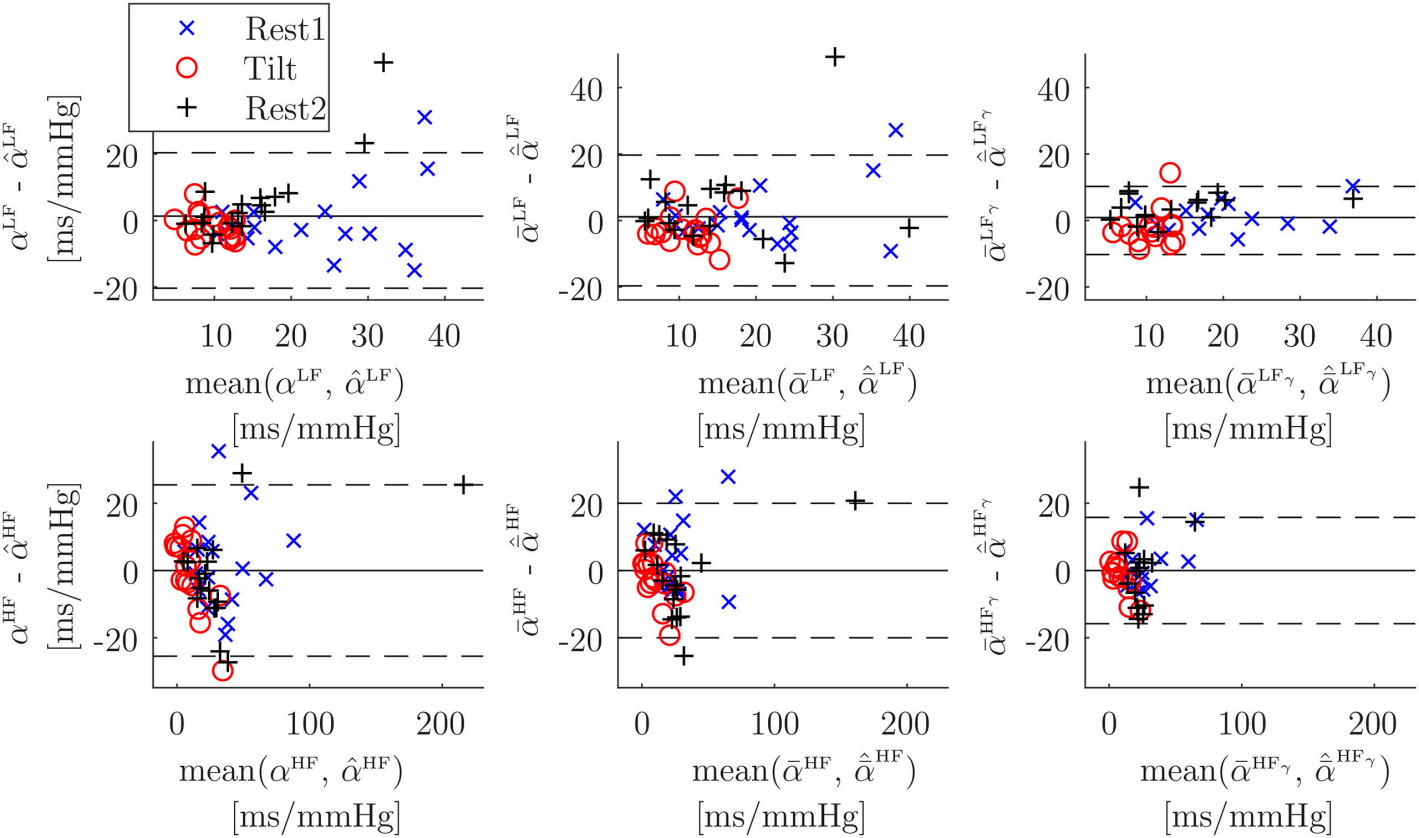
Bland-Altman plots of α vs. its multiple-linear-regression-based combination of surrogates $\hat{\alpha }$ (Welch-periodogram approach, first column), of $\bar{\alpha }$ vs. its multiple-linear-regression-based combination of surrogates $\hat{\bar{\alpha }}$ (time-frequency approach, second column), and of ${\bar{\alpha }}^{\gamma }$ vs. its multiple-linear-regression-based combination of surrogates ${\hat{\bar{\alpha }}}^{\gamma }$ (time-frequency coherence approach, third column). Note that scales are not the same for LF band (first row) than for HF band (second row).

The Bland-Altmant plot obtained from the multiple linear regression using all the studied indices is shown in Figure 7. The obtained limits of agreement were 1.31 ± 20.38, 2.61E–15 ± 25.48, 1.12 ± 19.68, –6.30E–15 ± 20.00, 0.89 ± 10.25, –6.85E–15 ± 15.80 ms/mmHg, for $\alpha_{\text{PUS}}^{\text{LF}}$, $\alpha_{\text{PUS}}^{\text{HF}}$, ${\bar{\alpha }}_{\text{PUS}}^{\text{LF}}$, ${\bar{\alpha }}_{\text{PUS}}^{\text{HF}}$, ${\bar{\alpha }}_{\text{PUS}}^{{\text{LF}}_{\gamma }}$, and ${\bar{\alpha }}_{\text{PUS}}^{{\text{HF}}_{\gamma }}$, respectively.

Table 2 shows the inter-subject median and interquartile ranges of α^{LF, HF}^ and $\alpha_{k}^{\left\{\text{LF},\text{HF}\right\}}$, for those indices which showed at least moderate Spearman's correlation coefficients (>0.50), and their relative increments (Δ). Significant differences (*p* < 0.05) of these indices between Tilt and rest stages are denoted with^*^. Similarly, the inter-subject median and interquartile ranges of medians of ${\bar{\alpha }}^{\left\{\text{LF},\text{HF}\right\}}$ and ${\bar{\alpha }}_{k}^{\left\{\text{LF},\text{HF}\right\}}$ are shown in Table 3, and interquartile ranges of medians of ${\bar{\alpha }}^{\left\{{\text{LF}}_{\gamma },{\text{HF}}_{\gamma }\right\}}$ and ${\bar{\alpha }}_{k}^{\left\{{LF}_{\gamma },{HF}_{\gamma }\right\}}$ are shown in Table 4.

**Table 2:**
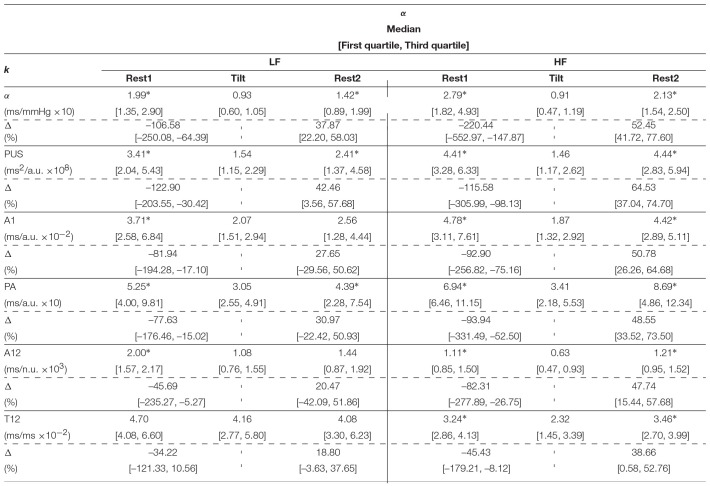
Inter-subject median and interquartile ranges of α^{LF, HF}^ and $\alpha_{k}^{\left\{\text{LF},\text{HF}\right\}}$.

Significant differences (p < 0.05) of these intra-subject medians between Tilt and rest stages are denoted with ^*^. In addition, the median and interquartile ranges of the relative increments (Δ) of these intra-subject medians between consecutive stages of the protocol (Rest1 and Tilt, and Tilt and Rest2) are also shown.

**Table 3:**
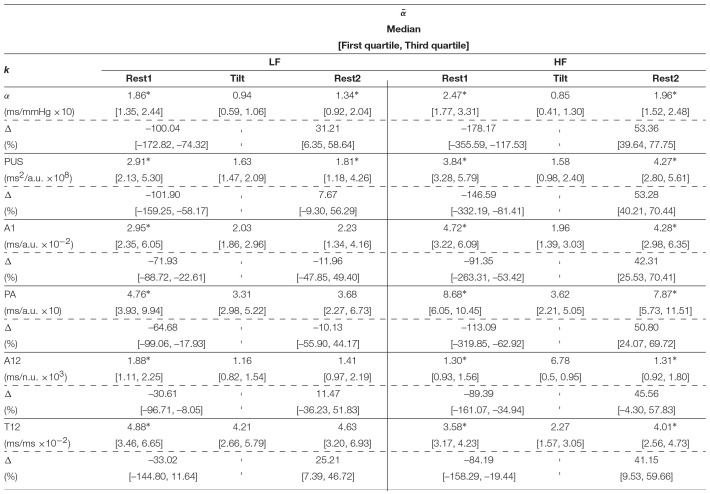
Inter-subject median and interquartile ranges of intra-subject median of ${\bar{\alpha }}^{\left\{\text{LF},\text{HF}\right\}}$ and ${\bar{\alpha }}_{k}^{\left\{\text{LF},\text{HF}\right\}}$.

Significant differences (p < 0.05) of these intra-subject medians between Tilt and rest stages are denoted with ^*^. In addition, the median and interquartile ranges of the relative increments (Δ) of these intra-subject medians between consecutive stages of the protocol (Rest1 and Tilt, and Tilt and Rest2) are also shown.

**Table 4:**
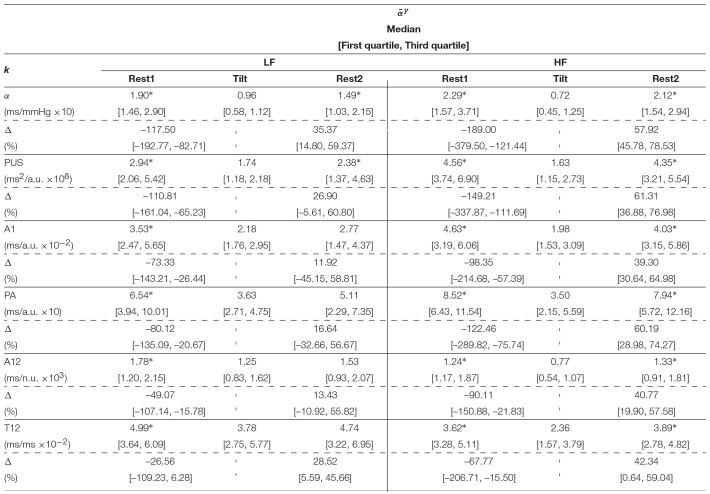
Inter-subject median and interquartile ranges of intra-subject median of ${\bar{\alpha }}^{\left\{{\text{LF}}_{\gamma },{\text{HF}}_{\gamma }\right\}}$ and ${\bar{\alpha }}_{k}^{\left\{{\text{LF}}_{\gamma },{\text{HF}}_{\gamma }\right\}}$.

Significant differences (p < 0.05) of these intra-subject medians between Tilt and rest stages are denoted with ^*^. In addition, the median and interquartile ranges of the relative increments (Δ) of these intra-subject medians between consecutive stages of the protocol (Rest1 and Tilt, and Tilt and Rest2) are also shown.

## 4. Discussion

Novel methods for measuring BRS using a PPG signal have been presented. They are based on surrogates of the α index, defined as the ratio of the power of RRV series and the power of SAPV series. In this work, PPV is used as a surrogate of RRV, and the SAPV is surrogated by different morphological features of the PPG pulse which have been related to BP in the literature. Some of these features are based on a novel PDA technique that has been presented in this paper. Many modeling functions have been applied to fit the PPG pulses in the literature. The novelty of the proposed PDA technique is that the used modeling function does not affect to the decomposition, as it is applied individually to the already extracted waves. It is worthy to note that the goal in this paper is not to obtain a very accurate measure of the studied morphological features, but in deriving a measure which is proportional to those features (as only their variability is needed). Keeping this in mind, a Gaussian function was used because it satisfies de goal while being a simple function that makes sense from the physiological point of view.

Three approaches were studied for estimating the α index from RRV and SAPV (or their surrogates): one based on Welch periodogram (α^{LF, HF}^), and two based on a time-frequency distribution which takes into account the non-stationarity of the protocol (Orini et al., 2012). This method redefines both LF and HF bands, making them time-varying following the dominant frequencies in such bands (${\bar{\alpha }}^{\left\{\text{LF},\text{HF}\right\}}$). Alternatively, this method computes a time-frequency coherence between the RRV and the SAPV (or their surrogates), and estimates the α index in restricted areas where the obtained coherence is statistically significant, i.e., in those areas evidencing that RRV and SAPV (or their surrogates) are coupled (${\bar{\alpha }}^{\left\{{\text{LF}}_{\gamma },{\text{HF}}_{\gamma }\right\}}$).

The correlation analysis shows how the PPG-based surrogates of α index track the changes of the conventional (ECG-and-BP-based) α index. Five out of the eleven SAPV surrogates leaded to α-index surrogates which obtained at least moderate correlation (>0.5). Those SAPV surrogates are, in order of cases getting the highest correlation: PUS, A1, PA, A12, and T12. Specifically, those α-index surrogates based on PUS obtained high correlation (>0.7) in all the cases. Those α-index surrogates based on A1 also obtained high correlation in four out of the six cases ($\alpha_{\text{A1}}^{\text{LF}}$, $\alpha_{\text{A1}}^{\text{HF}}$, ${\bar{\alpha }}_{\text{A1}}^{\text{HF}}$ and ${\bar{\alpha }}_{\text{A1}}^{{\text{HF}}_{\gamma }}$), while the remaining two (${\bar{\alpha }}_{\text{A1}}^{\text{LF}}$ and ${\bar{\alpha }}_{\text{A1}}^{{\text{LF}}_{\gamma }}$) were very close to obtain it (correlation was 0.69 in both cases).

Results regarding the BRS assessment are shown in Table 2 for the Welch-periodogram approach (α^{LF, HF}^), Table 3 for the time-frequency approach (${\bar{\alpha }}^{\left\{\text{LF},\text{HF}\right\}}$), and Table 4 for the time-frequency-coherence (${\bar{\alpha }}^{\left\{{\text{LF}}_{\gamma },{\text{HF}}_{\gamma }\right\}}$). The conventional α indices showed significant differences between both rest stages and Tilt within both LF and HF, and for the 3 approaches. The highest difference was observed between Rest1 and Tilt within HF band (-220.44% for α^HF^, -178.17% for ${\bar{\alpha }}^{\text{HF}}$, and -189.00% for ${\bar{\alpha }}^{{\text{HF}}_{\gamma }}$), while the smallest difference was observed between Rest2 and Tilt within LF band (37.87% for α^LF^, 31.21% for ${\bar{\alpha }}^{\text{LF}}$, and 35.37% for ${\bar{\alpha }}^{{\text{LF}}_{\gamma }}$).

The only SAPV surrogate which led to α-index surrogates showing the same behavior than the conventional α-index was PUS. None of the other SAPV surrogates led to α-index surrogates finding significant differences between Rest2 and Tilt within LF (the smallest observed change) with the exception of PA when using the Welch-periodogram approach ($\alpha_{\text{PA}}^{\text{LF}}$). However, PUS, A1, PA, and A12-based α-index surrogates found significant Rest1 and Tilt within both the LF and HF band, and between Rest2 and Tilt within the HF band, for the 3 approaches. The T12-based α-index surrogates also found these differences for both the time-frequency and the time-frequency-coherence approaches while they found significant differences only within HF band for the Welch-periodogram approach. In general, those PPG-based-α-index surrogates exploiting the pulse amplitude (PA, A1, and A12) obtained better results than those exploiting the pulse dispersion (PSTT, B1, C1, C2, T12, and T13) for BRS assessment, with the exception of T12. However, the best results were obtained for the index derived from PUS, which exploits both PPG amplitude and pulse dispersion. Another possible reason of the better results obtained by PUS is that it is measured at the beginning of the pulse, which would be the part related to a unique wave (main wave, before superposition of reflections) containing the BP information better expressed than the reflected waves.

The Bland-Altman plots (Figure 6) for PUS-based α-index surrogates (after converting units to ms/mmHg by a linear regression) are wider for HF (±60.90 ms/mmHg, ±40.49 ms/mmHg, and ±46.89 ms/mmHg, for Welch-periodogram, time-frequency, and time-frequency-coherence approaches, respectively) than for LF (±21.90, ±20.13, and ±18.43 ms/mmHg, for Welch-periogram-, time-frequency-, and time-frequency-coherence approaches, respectively). When combining all the PPG-based α-index surrogates by a multiple-linear regression, these limits of agreement are narrower, specially for within HF (±25.48, ±20.00, and ±15.80 mm/mmHg, for Welch-periogram-, time-frequency-, and time-frequency-coherence approaches, respectively). These results suggest that there is complementary information among the SAPV surrogates and thus, they could be combined for improving the α-index surrogate. However, this combination may require a calibration process which may be subject-specific in a final application. Further studies including data from same subjects during different days must be elaborated in order to explore techniques to combine the information of the different α-index surrogates.

Comparing the correlations obtained by the PUS-based α-index surrogates among the three α-index estimation approaches, the highest correlation within LF was obtained when using the Welch-periodogram approach (0.81), while the highest correlation within HF was obtained when using the time-frequency-coherence approach (0.81). However, given the intrinsic non-stationarity of the cardiovascular system, our recommendation is to use the time-frequency-coherence approach (Orini et al., 2012) because it takes into account the time-varying dominant frequencies and the strength of the coupling between RRV and SAPV (or their surrogates) and thus, its estimates are more related to the BRS than the estimates from the other two approaches.

Based on these results, our recommendation for PPG-based BRS assessment is ${\bar{\alpha }}_{\text{PUS}}^{\left\{{\text{LF}}_{\gamma },{\text{HF}}_{\gamma }\right\}}$. First, ${\bar{\alpha }}_{\text{PUS}}^{{\text{LF}}_{\gamma }}$ presented a significant decrease of more than 100% in median in tilt with respect to supine, which is in concordance with the decrease in reference ${\bar{\alpha }}^{{\text{LF}}_{\gamma }}$. Second, ${\bar{\alpha }}_{\text{PUS}}^{{\text{HF}}_{\gamma }}$ also presented a significant decrease in tilt with respect to supine, in this case around 2 times lower with respect to Rest1 (26.90%) than to Rest2 (61.31%), and these results are also in accordance to the reference ${\bar{\alpha }}^{{\text{HF}}_{\gamma }}$ (with 35.37% and 57.92%, respectively). It is worthy to note that the best α surrogate may be not derived from the best SAPV surrogate, because PPV was used as RRV surrogate while it is the sum of RRV and PAT variability (PATV) (Gil et al., 2010). Thus, for obtaining a exact surrogate for the ratio RRV/SAPV using PPV as numerator of the ratio, the best denominator is not exactly SAPV, but SAPV × (1+PATV/RRV).

These results support the potential value of the proposed index as a surrogate of BRS to monitor baroreflex impairment in certain applications. For example, in de Moura-Tonello et al. (2016) the square root of the RR and systolic BP series power (α index) at rest was significantly reduced (around 50%) in type 2 diabetes mellitus patients without cardiovascular autonomic neuropathy with respect to healthy controls of similar age and antropometric characteristics. In Ranucci et al. (2017) preoperative BRS was evaluated in 150 patients undergoing coronary surgery and related to postoperative complications such as atrial fibrillation, renal function impairment and low cardiac output syndrome. The α index was significantly lower (around 30% in median) in patients experiencing postoperative acute kidney dysfunction, as well as in patients with low cardiac output state (around 50% in median). However, clinical studies have to be elaborated in order to evaluate the proposed indices in different applications. To the best of our knowledge, this is the first time that these indices are studied for BRS assessment, so healthy volunteers with presumably efficient baroreflex were used in order to observe actual changes along the protocol. Different results may be obtained with patients of different diseases, specially taking into account that coherence is reduced in heart disease patients.

Results reported in this work suggest that BRS can be assessed with high correlation by only a PPG signal based on PPV (as RRV surrogate), and PPG-amplitude-based and/or PPG-dispersion-based features (as SAPV surrogates), being PUS the most convenient SAPV surrogate for BRS assessment. The PPG signal recording is simple, economical, and comfortable for the subject. Moreover, PPG signal can be acquired in many places of the body. Thus, these results are very interesting for ambulatory scenarios and for wearable devices. Future studies may include an surrogate of the α index using a combination of different PPG-based SAPV surrogates, specially amplitude- and dispersion-based features.

## Ethics Statement

This study was carried out in accordance with the recommendations of Comité Ètico de Investigación Clínica de Aragón (CEICA) with written informed consent from all subjects. All subjects gave written informed consent in accordance with the Declaration of Helsinki. This study was exempt from approval of the ethical committee because no new data were registered for its development.

## Author Contributions

JL and RB wrote the first draft in the manuscript preparation. All authors participated in the design of the proposed methods, as well of the statistical analysis for their evaluation, reviewed, and critiqued the manuscript preparation.

### Conflict of Interest Statement

The authors declare that the research was conducted in the absence of any commercial or financial relationships that could be construed as a potential conflict of interest.

## Abbreviations

BP: Blood pressure
BRS: Baroreflex sensitivity
ECG: Electrocardiograml
HF: High frequency
HR: Heart rate
HRV: Heart rate variability
LF: Low frequency
PA: Pulse amplitude
PAT: Pulse arrival time
PATV: Pulse arrival time variability
PDA: Pulse decomposition analysis
PEP: Pre-ejection period
PPG: Pulse photoplethysmography
PPV: Pulse-to-pulse variability
PSTT: Pulse slope transit time
PTT: Pulse transit time
PUS: Pulse up-slope
PW: Pulse width
RRV: RR interval variability
SAPV: Systolic arterial pressure variability
SD: Standard deviation.
